# Changes of peripheral T cell subsets in melanoma patients with immune-related adverse events

**DOI:** 10.3389/fimmu.2023.1125111

**Published:** 2023-04-12

**Authors:** Benjamin Müller, Anne Bärenwaldt, Petra Herzig, Alfred Zippelius, Lara Valeska Maul, Viviane Hess, David König, Heinz Läubli

**Affiliations:** ^1^Laboratory for Cancer Immunotherapy and Immunology, Department of Biomedicine, University of Basel, Basel, Switzerland; ^2^Division of Oncology, University Hospital Basel, Basel, Switzerland; ^3^Department of Dermatology, University Hospital Basel, Basel, Switzerland

**Keywords:** cancer immunotherapy, PD-1, CTLA-4, immune checkpoint, colitis, hepatitis

## Abstract

**Introduction:**

Immunotherapies have improved the prognosis of many cancer patients including patients with advanced melanoma. Immune checkpoint receptors including CTLA-4 and PD-1 have been established as main therapeutic targets for immunotherapy of melanoma. Although monotherapy is effective in melanoma patients, a dual therapy approach has been shown to be most effective. Dual checkpoint blockade, however, increases substantially the risk for immune-related adverse events (irAEs).

**Methods:**

In this study, we characterized peripheral immune cell subsets in patients with anti-PD-1 monotherapy and with dual immune receptors blockade targeting PD-1 and CTLA-4.

**Results:**

We found differences in peripheral T cells between patients who developed severe immune-related side effects and patients with mild irAEs. We identified several mainly changes in CD8^+^ T cell subsets in patients with severe irAE under dual PD-1 and CTLA-4 blockade.

**Discussion:**

This work suggests that peripheral immune cell dynamics could be associated with severe immune-related side effects in patients receiving immune checkpoint inhibitors. These changes could be used as future biomarkers in early diagnosis of irAEs.

## Introduction

Advances in our understanding of anti-tumor immunity have led to the development of new cancer immunotherapies (1). Current approaches used in the clinic includes the blockade of inhibitory receptors like PD-1, CTLA-4 and LAG-3 on the surface of immune cells, especially on T cells (1). This immune checkpoint inhibitors (ICI) substantially improved the prognosis of patients with immunogenic cancers such as melanoma, non-small lung cancer or urthelial cancers (2, 3). However, the introduction of ICI also generated new immune-mediated side effects or immune-related adverse events (irAEs) that are a significant concern for these patients (4–6). Several mechanisms including antigen cross-presentation, auto-antibody formation, and cytokine-mediated inflammation have been identified as potential mechanism (4–7). Changes of peripheral immune cells have been associated with anti-tumor response but changes in patients with severe irAE are not yet well studied (8, 9). Here, we analyzed immune cell changes in the peripheral blood of patients receiving PD-1 mono-blockade and PD-1/CTLA-4 dual blockade with a focus on peripheral T cell changes in patients developing irAEs.

## Materials and methods

### Patient cohort and definition of severe irAEs

Patients with melanoma treated with ICI at the Oncology division of the University Hospital in Basel were invited to participate in this cohort study. Inclusion criteria for cohort 1 were: monotherapy with PD-1 blocking antibody (pembrolizumab or nivolumab), for cohort 2 combination ICI treatment with PD-1 and CTLA-4 blocking antibodies (nivolumab and ipilimumab). Clinical data was collected from the electronic hospital data base. Severe irAEs were defined as AEs that led to permanent discontinuation of ICI therapy, all other irAEs were deemed “mild”.

### Peripheral blood sampling

Ethics approval was obtained from the local ethical committee to analyze blood samples (Northwest and Central Switzerland Ethics Committee EK321/10). Peripheral blood mononuclear cells (PBMCs) were collected, isolated by a Ficoll density gradient and frozen for later analysis. Blood was obtained before the start of the ICB and 4-6 weeks after start of ICB. Isolated PBMCs were cryopreserved by adding 10% DMSO containing fetal calf serum.

### Multicolor flow cytometry

We established and validated flow cytometry panel (surface marker: CD45RO, CD14, CD11c, CD127, CD11b, CD335, CD25, HLA-DR, CD8, CD16, CD56, CD3, live/dead, CD123, CD19, CD4, PD-1, and intracellular marker: FoxP3, Ki-67). A single-stain control was made using VersaComp Antibody Capture Beads for each fluorochrome (Beckman Coulter, USA). Cells were heated at 95 degrees for 5min for single-stain control of dead cells. Dead cells were stained with live/dead blue. Fluorophore-conjugated antibodies were diluted in FACS buffer (PBS, 2% FBS, 0.5 mM EDTA). Patient samples were thawed at 37 degrees and washed with FACS buffer. All cells were incubated for 20min on ice with Live/dead Blue in PBS, followed by 20min incubation with anti-human Fc block (Invitrogen, USA) to prevent unspecific antibody binding. Afterwards the cell suspension was stained with a surface fluorophore-conjugated antibody solution for 30min on ice. After incubation cells were treated with a fix/perm solution (eBioscience, USA) before staining with the an intracellular antibody mix. Cells were resuspended in FACS buffer and fluorescence measured with a Cytek Aurora machine (Cytek Biosciences Inc., USA). The acquired data was analyzed using FlowJo 10.3 (TreeStar Inc, USA) after singlet- (FSC-A vs. FSC-H and SSC-A vs. SSC-W) and live/dead cell discrimination (gating strategy: **Supplementary Figures 1, 2**).

### Statistical analysis

Patient characteristics are presented in a descriptive manner. For the statistical analysis of the acquired Aurora data GraphPad Prism was used (Version 9). To compare the two patient groups (severe and mild irAEs) we used a non-parametric T test (Mann-Whitney t-test, p < 0.05 was considered as statistically significant).

## Results

### Patient cohorts

We collected blood samples of two cohorts of patients. In the first cohort, we included melanoma patients receiving PD-1 blocking monotherapy with nivolumab or pembrolizumab (**Table 1**). In the second cohort, we included melanoma patients receiving dual immune checkpoint blockade with nivolumab and ipilimumab targeting PD-1 and CTLA-4 (**Table 2**). In each of the two cohorts we divided the patients into patients that developed severe irAEs leading to cessation of the ted ICI therapy and patients with only mild side effects. In the monotherapy group, we observed 3 patients with irAEs that led to the discontinuation of the ICI therapy mainly due to the type of irAE including myocarditis, colitis and arthritis (**Table 1**). In cohort 2, six patients had to stop the treatment with dual ICI therapy with ipilimumab and nivolumab. Colitis and hepatitis were the most prevalent irAE observed in this cohort that led to discontinuation (**Table 2**). In general, patients receiving monotherapy had less advanced disease and were often treated in an adjuvant setting (**Table 1**). Patients in the cohort receiving dual ICI therapy had more often advanced stage IV disease (**Table 2**). No direct association was seen between relapse and irAE (**Table 1**). This is probably due to the low number of patients involved in this correlative study.

**Table 1:** Patients receiving PD-1 blocking monotherapy.

sex	tumor	age	stage	treatment	adverse event	IrAE Grade**	IrAE clinical severity	outcome
female	melanoma	59	IIIC	Pembro	arthritis	G2	mild	relapse
male	melanoma	67	IV (resected)	Nivo	hypothyroidism	G2	mild	relapse
male	melanoma	56	IIIC	Pembro	sarcoid-like reaction (lymphadenopathy)	G1	mild	relapse
male	melanoma	76	IIIC	Pembro	hypothyroidism	G1	mild	relapse
male	melanoma	48	IIIC	Nivo	na	na	mild	relapse
male	melanoma	78	IV	Nivo	hypothyroidism	G1	mild	PR
male	melanoma	85	IV	Pembro	pancreatitis	G2	mild	PR
male	melanoma	63	IIID	Nivo	arthritis	G2	severe*	relapse
male	melanoma	74	IIIA	Pembro	myocarditis	G1	severe*	relapse
male	melanoma	72	IIIC	Nivo	colitis	G2	severe*	relapse

* severe = immunosuppressive treatment necessary and permanent stop of ICIs.

**grading according to guidelines.

**Table 2:** Patients receiving PD-1 and CTLA-4 ICB.

sex	tumor	age	stage	treatment	adverse event	IrAE Grade**	IrAE clinical severity	outcome
female	melanoma	53	IIIC	Nivo/Ipi	duodenitis	G2	mild	relapse
female	melanoma	34	IV	Nivo/Ipi	colitis/enteritis	G1	mild	PD
male	melanoma	75	IV	Nivo/Ipi	hypophysitis	G2	mild	PR
male	melanoma	46	IV	Nivo/Ipi	hepatitis	G2	mild	SD
male	melanoma	72	IV	Nivo/Ipi	colitis	G3	severe*	PD
female	melanoma	54	IIB (localrelapse)	Nivo/Ipi	colitis	G3	severe*	PR
male	melanoma	85	IV	Nivo/Ipi	colitis	G2	severe*	PD
male	melanoma	43	III-IV	Nivo/Ipi	hepatitis	G4	severe*	CR
female	melanoma	58	IV	Nivo/Ipi	hepatitis	G2	severe*	PR
male	melanoma	51	IV	Nivo/Ipi	hepatitis	G3	severe*	PR

* severe = immunosuppressive treatment necessary and permanent stop of ICIs.

**grading according to guidelines.

### T cell changes in patients with severe irAEs receiving anti-PD-1 monotherapy

**Figure 1:**
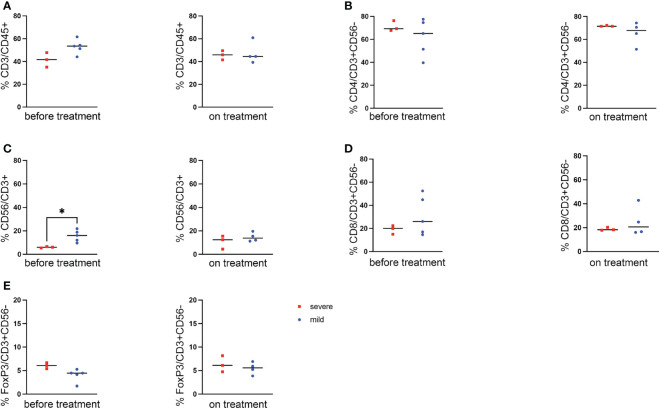
Changes of lymphocyte populations in patients treated with PD-1 monoblockade. **(A)** Comparison of mean percentage of CD3^+^ cells in lymphocytes between the two patient groups of cohort 1 (severe vs mild) on timepoint one and timepoint two (before treatment and 4-6 weeks after start). **(B–D)** CD4^+^ **(B)**, CD56^+^ **(C)** and CD8^+^ T cells **(D)** at different time points compared between patients developing severe irAEs and patients with only mild side effect. **(E)** Frequency of FoxP3^+^ regulatory T cells in the two groups of patients. Statistical analysis was performed by a T test. * p<0.05.

**Figure 2:**
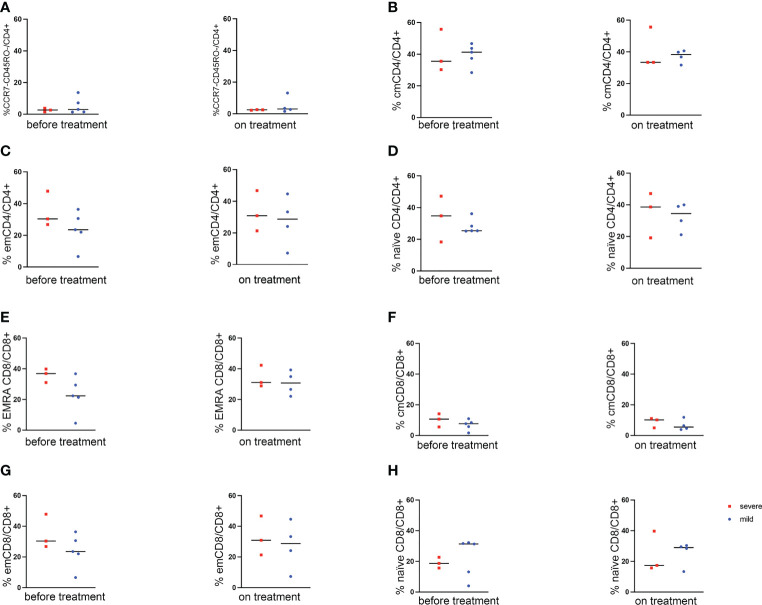
Analysis of lymphocyte subpopulations in patients treated with PD-1 monoblockade. Comparison of mean percentage of subpopulations of lymphocytes between the two patient groups of cohort 1 (severe vs mild) before the treatment and on the treatment. **(A–D)** CD4^+^ T cell subpopulations: **(A)** CD4^+^ CCR7^-^ CD45RO^-^ cells. **(C)** effector memory (em, CD4^+^ CCR7^-^ CD45RO^+^ cells). **(B)** central memory (cm, CD4^+^ CCR7^+^ CD45RO^+^ cells). **(C)** effector memory (em, CD4^+^ CCR7^-^ CD45RO^+^ cells). **(D)** naïve (CD4^+^ CCR7^+^ CD45RO^-^ cells). **(E–H)** CD8^+^ T cell subpopulations: **(E)** T EMRA (CD8^+^ CCR7^-^ CD45RO^-^ cells). **(F)** central memory (cm, CD8^+^ CCR7^+^ CD45RO^+^ cells). **(G)** effector memory (em, CD8^+^ CCR7^-^ CD45RO^+^ cells). **(H)** naïve (CD8^+^ CCR7^+^ CD45RO^-^cells).

To analyze the immune cell composition of patients with mild and severe irAEs after ICB, we stained PBMCs before treatment start and at time of irAE development and analyzed them by flow cytometry. In patients receiving PD-1 blocking monotherapy, no differences in CD3^+^ T cells were observed between patients with mild or severe irAEs (**Figure 1A**). In addition, no or only minimal changes were seen in T cell subsets including CD4^+^, CD8^+^ T cells, CD56^+^ T cells and FoxP3^+^ regulatory T cells (**Figures 1B–E**). CD56^+^ T cells were slightly increased in patients with only mild side effects before starting ICI therapy (**Figure 1C**). No differences were observed over time (time point 1 before the start and time point 2 taken 4-6 weeks after start of ICI therapy). Further characterization of T cell subsets showed no significant difference in both CD4 and CD8 compartments between patients with severe irAEs or mild irAEs (**Figures 2A–H**). In addition, no differences were observed in peripheral C11c^+^ dendritic cells, CD123^+^ plasmacytoid dendritic cells, CD19^+^ B cells, or CD14^+^ myeloid cells (**Supplementary Figure 3**). Moreover, no differential expression of Ki67, CD25, and PD-1 was demonstrated (**Supplementary Figures 4, 5**). Taken together, no clear differences of T cell subsets in the peripheral blood of patients treated with PD-1 blocking monotherapy were found.

### Changes in T cell subsets in patients with severe irAEs receiving dual immune checkpoint blockade

**Figure 3:**
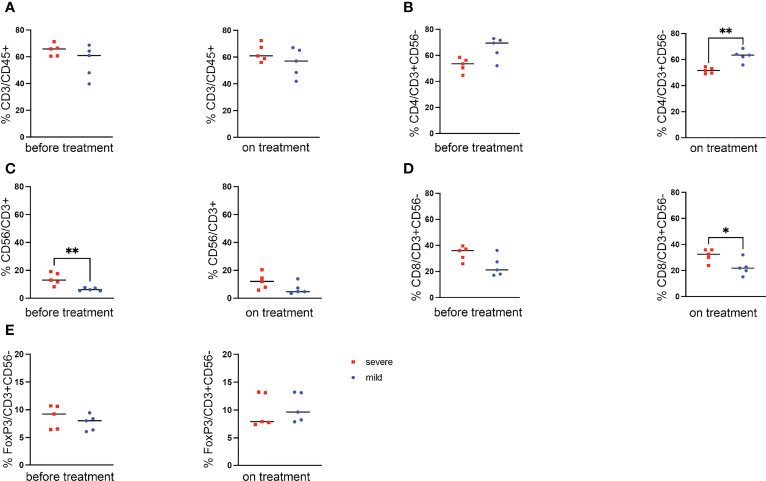
Changes of lymphocyte populations in patients treated with PD-1 and CTLA-4 dual ICB. **(A)** Comparison of mean percentage of CD3^+^ cells in lymphocytes between two patient groups of cohort 1 (severe vs mild) before treatment and on the treatment (before treatment and 4-6 weeks after start). **(B–D)** CD4^+^ **(B)**, CD56^+^ **(C)** and CD8^+^ T cells **(D)** at different time points compared between patients developing severe irAEs and patients with only mild side effect. **(E)** Frequency of FoxP3^+^ regulatory T cells in the two groups of patients. Statistical analysis was performed by a T test. ** p<0.01.

**Figure 4:**
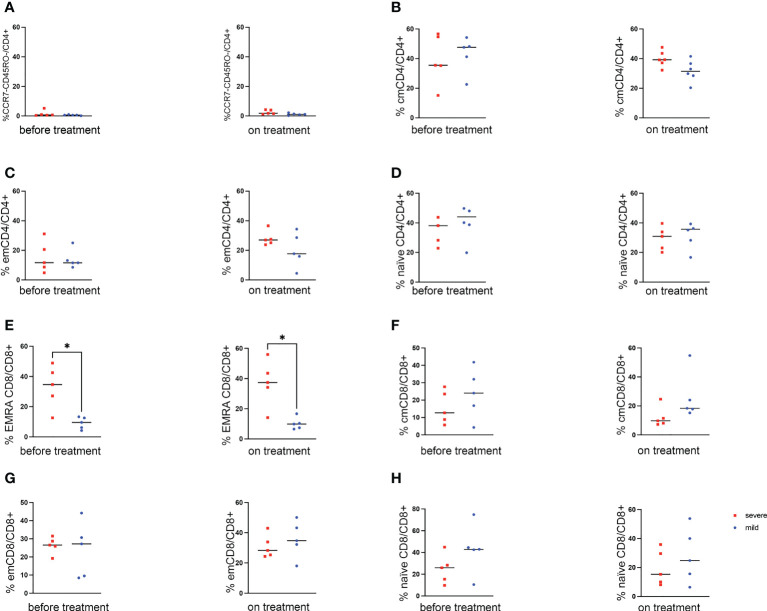
Analysis of lymphocyte subpopulations in patients treated with PD-1 and CTLA-4 dual ICB. Comparison of mean percentage of subpopulations of lymphocytes between the two patient groups of cohort 1 (severe vs mild) before the ICB and on treatment. **(A–D)** CD4^+^ T cell subpopulations: **(A)** CD4^+^ CCR7^-^ CD45RO^-^ cells. **(C)** effector memory (em, CD4^+^ CCR7^-^ CD45RO^+^ cells). **(B)** central memory (cm, CD4^+^ CCR7^+^ CD45RO^+^ cells). **(C)** effector memory (em, CD4^+^ CCR7^-^ CD45RO^+^ cells). **(D)** naïve (CD4^+^ CCR7^+^ CD45RO^-^ cells). **(E–H)** CD8^+^ T cell subpopulations: **(E)** T EMRA (CD8^+^ CCR7^-^ CD45RO^-^ cells). **(F)** central memory (cm, CD8^+^ CCR7^+^ CD45RO^+^ cells). **(G)** effector memory (em, CD8^+^ CCR7^-^ CD45RO^+^ cells). **(H)** naïve (CD8^+^ CCR7^+^ CD45RO^-^ cells). * p<0.05 by T test.

During PD-1 blocking monotherapy, only few patients developed severe irAEs. In contrast, patients treated with dual ICI therapy develop severe irAEs more often. Therefore, we further analyzed immune cell changes in the peripheral blood of these patients, comparing severe irAEs that led to the discontinuation of dual ICI therapy with mild irAE patients. No changes in percentage of T cells were observed in the two groups and also over time (**Figure 3A**). However, a decreased frequency of CD4^+^ T cells and Increased frequency of CD56^+^ T cells and CD8+ T cells before and after the start of the treatment was found (**Figures 3B–D**). No changes and differences were observed in FoxP3^+^ regulatory T cells (**Figure 3E**). Additional analysis of T cell subsets in this cohort of patients under dual ICI therapy showed no changes (**Figures 4A–H**), except for the CD8^+^ effector memory cells re-expressing CD45RA (EMRA, **Figure 4E**). An increased frequency of EMRA CD8^+^ T cells was seen before and after start of dual ICB with PD-1 and CTLA-4 blocking antibodies (**Figure 4E**). No major changes were seen between patients with mild and severe irAEs in other cell types (**Supplementary Figure 3**). We observed an increase in Ki67^+^ CD8 T cells after starting the treatment in patients experiencing severe irAE (**Supplementary Figures 4, 5**).

In patients with dual ICI therapy targeting PD-1 and CTLA-4, changes in peripheral T cells in patients with severe irAE could be detected that where already present before start of the treatment and remained during the course of treatment.

## Discussion

In this work, we identified differences in the peripheral immune cells, in particular T cells, between melanoma patients receiving PD-1 and CTLA-4 directed ICI therapy who developed irAEs. Only minimal changes were observed in patients under PD-1 blocking monotherapy. We found some slight differences of CD56^+^ NKT cells in patients with severe irAEs leading to ICI discontinuation but the sample size is limited. It has been shown that NKT cells can play a role in anti-tumor immunity and that ICI therapy therefore also promotes NKT cell activity (10, 11). NKT cells are a unique subset of T cells that function as crosslink between innate and adaptive immunity (10, 12). NKT cells modulate immune response of several other immune cells such as tumor-specific T cells and effector NK cells at an early stage of tumorigenesis (10, 11). In the cohort of patients receiving dual ICI therapy, we found also an association of increased NKT cells and the frequency of severe irAEs. The exact implication, however, of NKT cells and occurrence of irAEs will require further studies and confirmation.

In the cohort of patients receiving PD-1 and CTLA-4 blocking antibodies, we have seen additional changes in the peripheral T cell subsets of patients that experienced severe irAEs leading to treatment discontinuation. We have observed a decreased CD4^+^ to CD8^+^ T cell ratio in patients with severe irAEs, and in particular an increased frequency of CD8^+^ EMRA T cells defined as CD8+ CD45RO- CCR7- cells. These T cells were previously associated with various diseases including Alzheimer disease (13), amyotrophic lateral sclerosis (14), virus infection (15–17), smoking-associated T cell dysfunction (18), and cancer (9, 19). In HIV infections, CD8^+^ EMRA T cells were associated with better outcome and control of early viremia (16). In another study, Hess and colleagues found an improved control of HIV in patients that have expanded this EMRA population (15). EMRA CD8^+^ T cells have been described as highly cytotoxic (20). It is therefore conceivable that this population could mediate stronger immune reactions in patients receiving ICI therapy. Recent data that characterized T cells in patients with cancer by have defined various T cell subsets including T EMRA CD8^+^ T cells in the peripheral blood (21). In a recent work, Bukhari and colleagues have found non-statistical differences of EMRA CD8^+^ T cells in patients developing immune-mediated arthritis, although the authors have only analyzed baseline differences (9). Our findings suggest that peripheral immune cell dynamics in the blood could be associated with severe immune-related side effects in melanoma patients receiving ICB. We observed changes of EMRA CD8^+^ T cells in the blood, which could be different compared to what is happening in the peripheral tissue including tumor sites. Taken together, the function of this peripheral EMRA T cell population is not yet clear and further investigations are needed to understand the role of this subset and the potential use as a biomarker.

We are aware of the limitation of our analysis due to the small sample size. Larger and independent cohorts are required. In addition, a more homogenous patient populations could help the reveal potential biomarkers to predict irAEs. In a recent cohort, peripheral blood cells were analyzed by single cell transcriptomics (22). Interestingly, T cell phenotypes associated with irAEs were also organ specific. Other studies have analyzed immune cells in peripheral blood of patients receiving ICI (8, 23–25). Some of these analyses have also focused on the development of irAEs (25). Similar to our findings, these groups have observed an increase of Ki67^+^ CD8^+^ T cells after start of the treatment (25). Our data independently confirms therefore changes observed by other groups in larger cohorts and systemic changes in immune cells could be potentially used for the early recognition of patients that develop irAEs.

## Data availability statement

The original contributions presented in the study are included in the article/**Supplementary Material**. Further inquiries can be directed to the corresponding author.

## Ethics statement

The studies involving human participants were reviewed and approved by Northwest and Central Switzerland Ethics Committee EK321/10. The patients/participants provided their written informed consent to participate in this study.

## Author contributions

HL, VH, and DK conceived the project idea. PH, DK, LM, and AZ coordinated the patient care and sample collection. AB designed the Aurora Panel and supervised experiments. BM performed experiments. BM and HL wrote the manuscript. All authors contributed to the article and approved the submitted version.
