# Supplementary material for: Changes of peripheral T cell subsets in melanoma patients with immune-related adverse events
Source: Front Immunol. 2023 Apr 12;14:1125111. doi: 10.3389/fimmu.2023.1125111 (PMC10130408; doi:10.3389/fimmu.2023.1125111)
Supplement: Supplementary file 1 [file DataSheet_1.docx]

24 March 2023

**Supplementary Data**

**Changes of peripheral T cell subsets in melanoma patients with immune-related adverse events**

Benjamin Müller, Anne Bärenwaldt, Petra Herzig, Alfred Zippelius, Lara Maul, Viviane Hess, David König, and Heinz Läubli

**
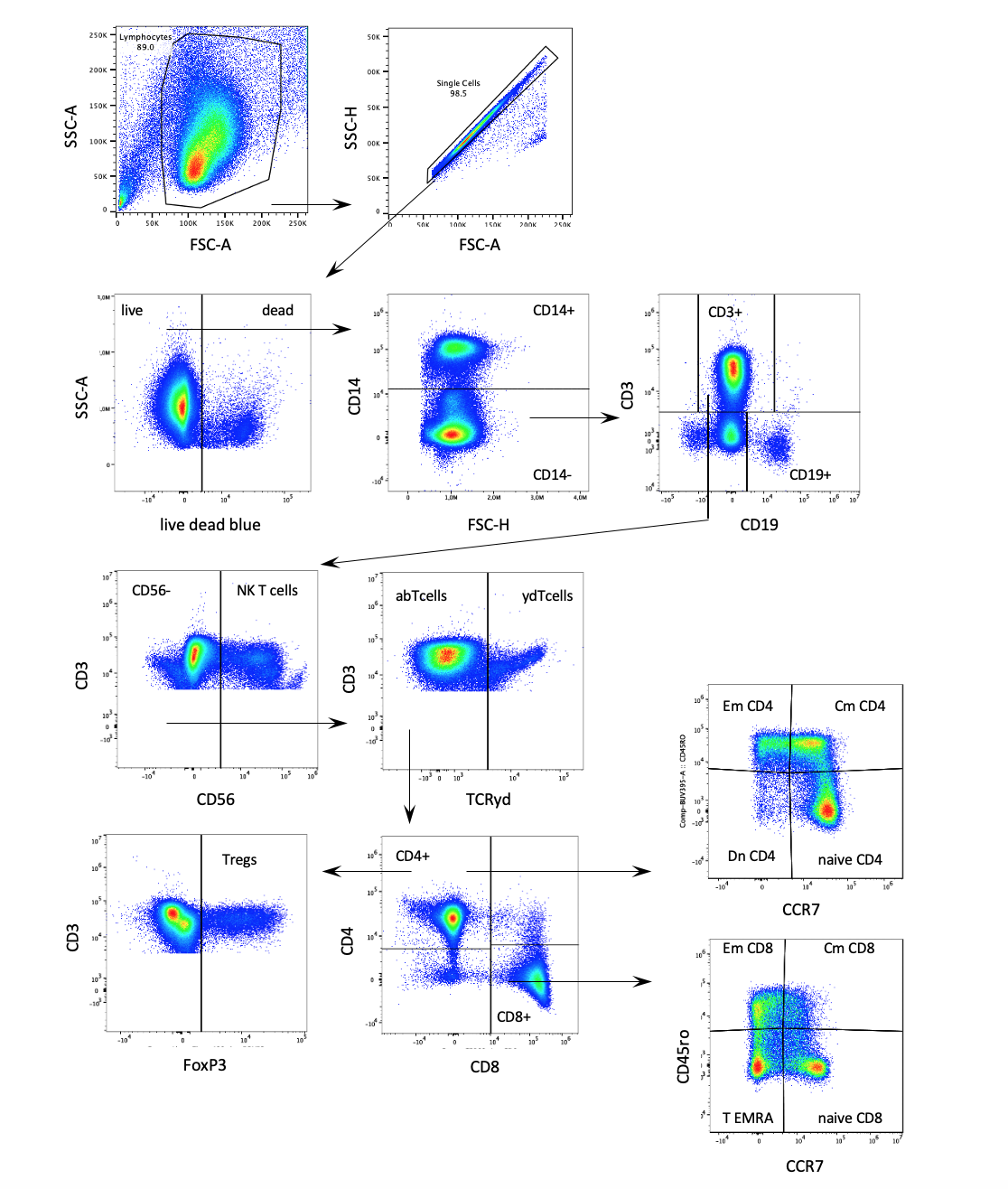
Supplementary Figure and Table.**

**Supplementary Figure 1 Gating strategy for T cell subset analysis.**


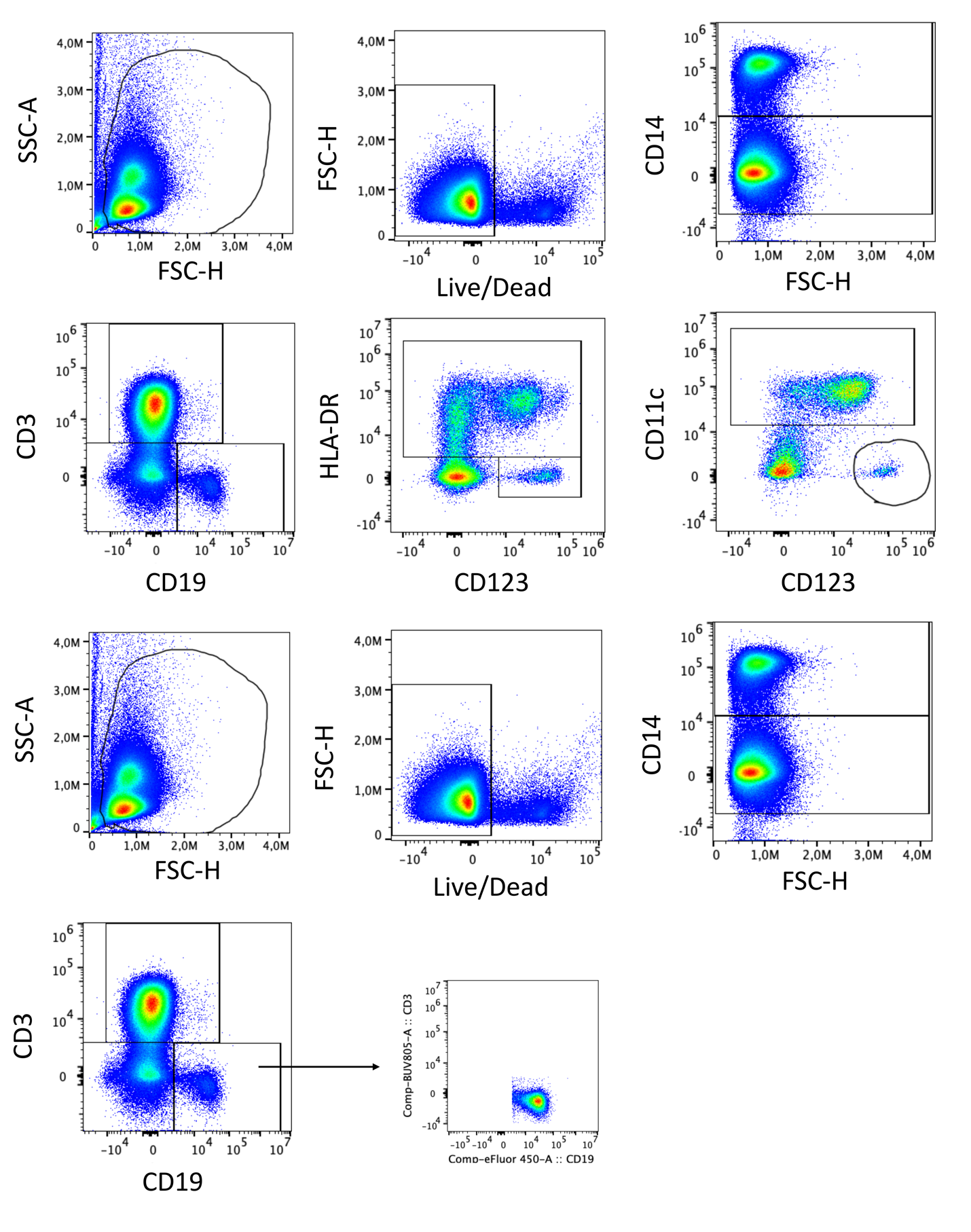


**Supplementary Figure 2 Gating strategy for cell frequencies.**


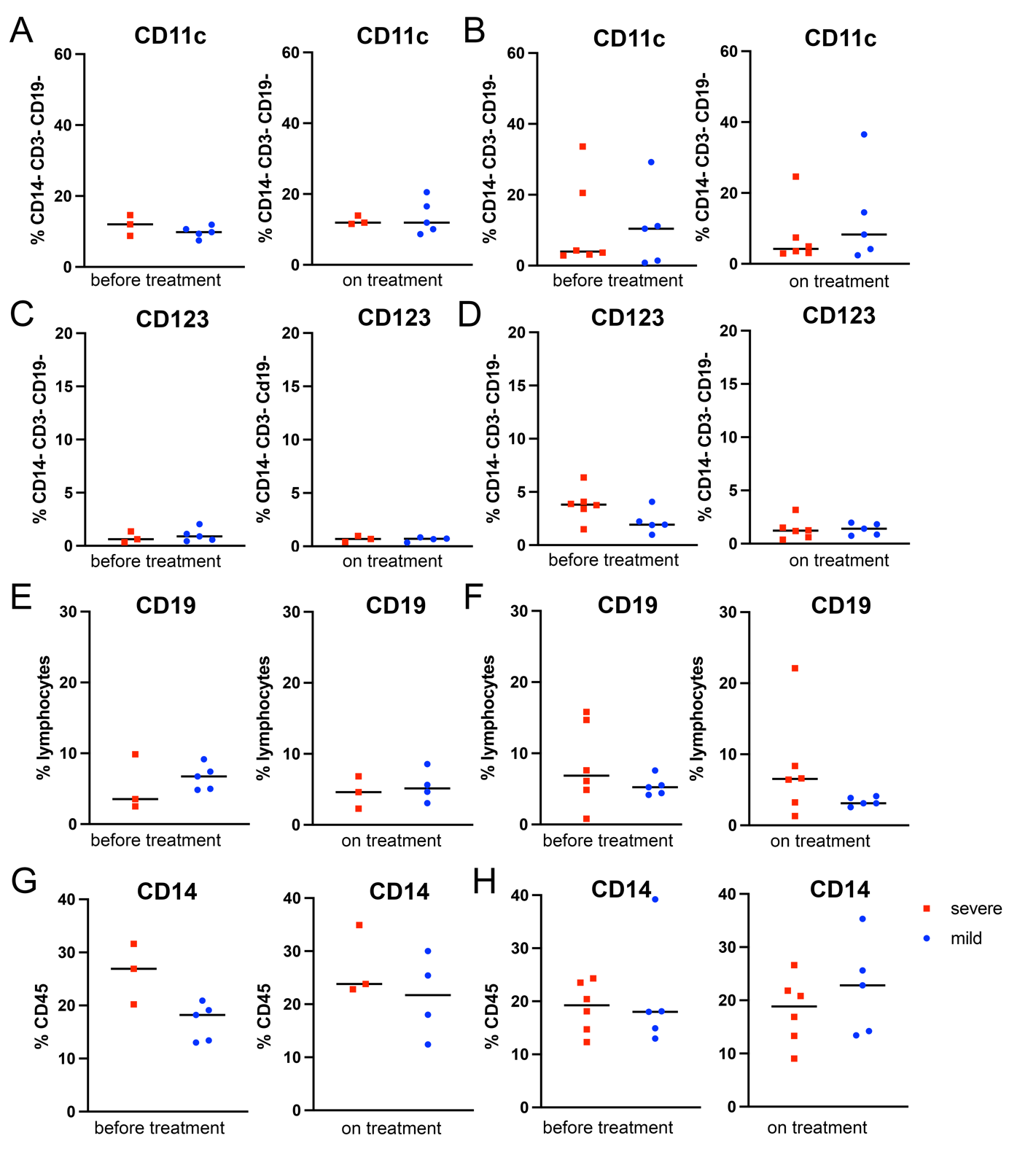


**Supplementary Figure 3 Analysis of non-T cell cells in the peripheral blood of patients treated with ICI. A**, CD11c positive dendritic cells in patients of cohort 1 before and on treatment. **B**, CD11c positive dendritic cells in patients of cohort 2 before and on treatment. **C**, CD123 plasmacytoid dendritic cells positive dendritic cells in patients of cohort 1 before and on treatment. **D**, CD123 plasmacytoid dendritic cells positive dendritic cells in patients of cohort 2 before and on treatment. **E**, CD19 B cells positive dendritic cells in patients of cohort 1 before and on treatment. **F**, CD19 B cells positive dendritic cells in patients of cohort 2 before and on treatment. **G**, CD14 myeloid cells positive dendritic cells in patients of cohort 2 before and on treatment. **H**, CD14 myeloid cells positive dendritic cells in patients of cohort 1 before and on treatment.


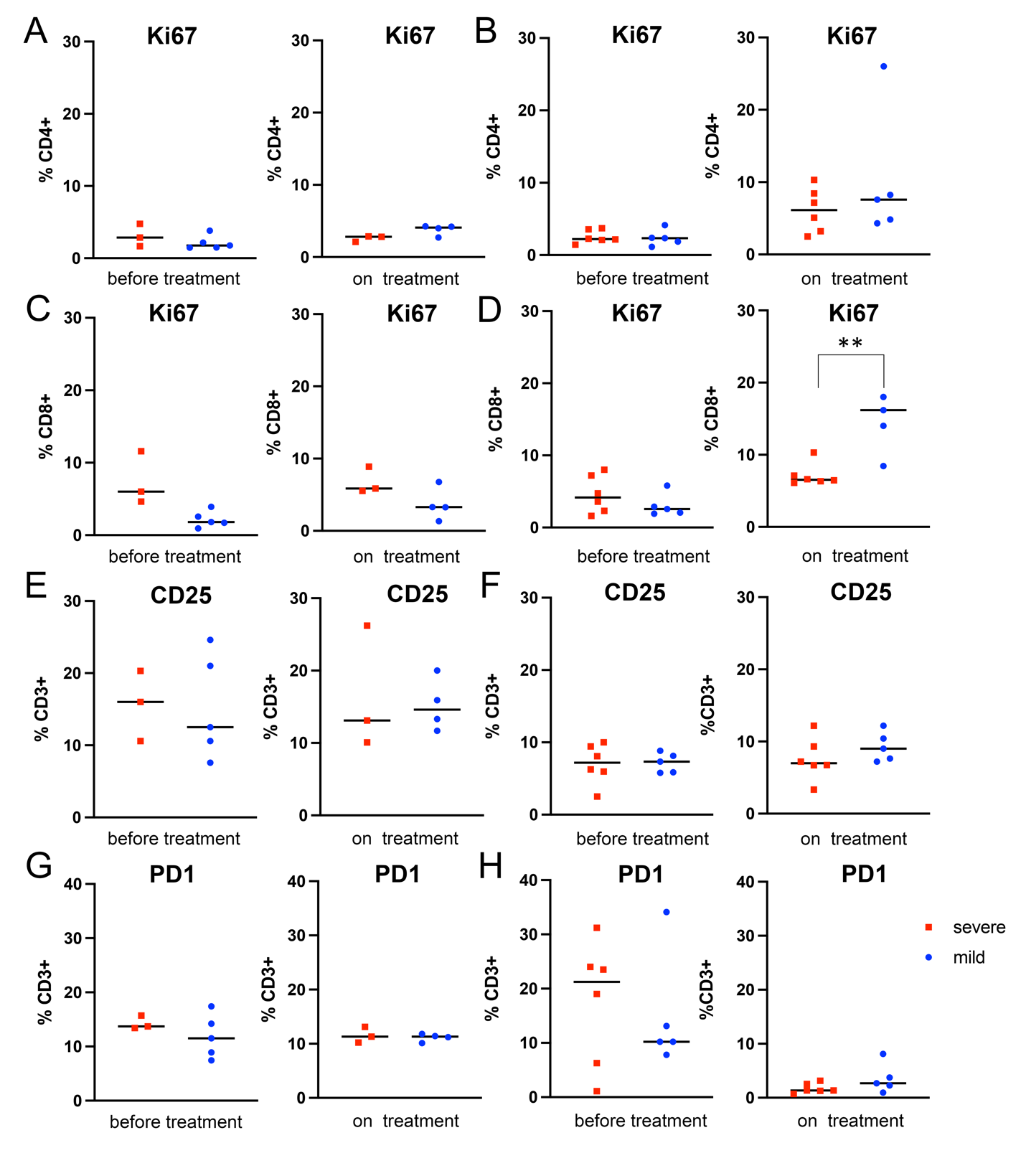
**Supplementary Figure 4 Analysis of T cell activation in patients. A**, Percentage of Ki67 positive CD4 T cells in patients of cohort 1 before and after the treatment. **B**, Percentage of Ki67 positive CD4 T cells in patients of cohort 2 before and after the treatment. **C**, Percentage of Ki67 positive CD8 T cells in patients of cohort 1 before and after the treatment. **D**, Percentage of Ki67 positive CD8 T cells in patients of cohort 2 before and after the treatment. **E**, Percentage of CD25 positive T cells in patients of cohort 1 before and after the treatment. **F**, Percentage of CD25 positive T cells in patients of cohort 2 before and after the treatment. **G**, Percentage of PD1 positive T cells in patients of cohort 1 before and after the treatment. **H**, Percentage of PD1 positive T cells in patients of cohort 1 before and after the treatment.

**
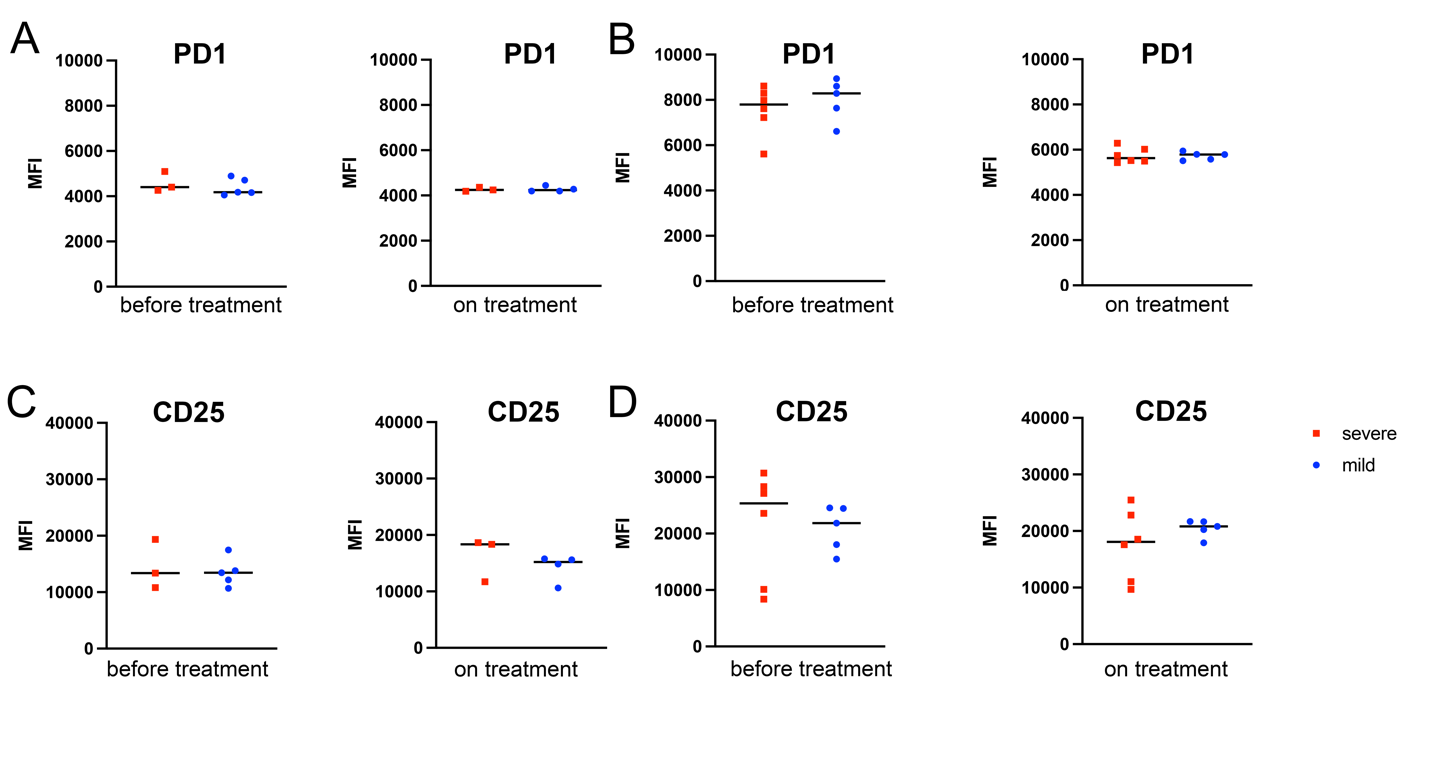
Supplementary Figure 5 Analysis of T cell activation in patients. A**, MFI of on CD3 T cells in patients of cohort 1 before and after the treatment. **B**, MFI of PD-1 on CD3 T cells in patients of cohort 2 before and after the treatment. **C**, MFI of CD25 on CD3 T cells in patients of cohort 1 before and after the treatment. **D**, MFI of CD25 on CD3 T cells in patients of cohort 2 before and after the treatment.

| **marker** | **Fluorochrome** | **marker** | **Fluorochrome** |
| --- | --- | --- | --- |
| CD45RO | BUV395 | FoxP3 | A488 |
| CD14 | BUV563 | Ki-67 | AF532 |
| CD56 | BUV661 | CD4 | NovaBlue 610 |
| CD11c | BUV737 | CD8 | CF568 |
| CD3 | BUV805 | CD25 | PE-Fire700 |
| dead cells | Live/Dead UV | HLA-DR | PE-Fire810 |
| CXCR3 | PerCP-Cy5.5 | PD-1 | APC |
| CD123 | SuperBright436 | CCR7 | APC-Fire810 |
| CD19 | eFluor 450 | CD16 | AF700 |
| CD11b | BV650 | CD27 | APC-Cy7 |

**Supplementary Table 1** Markers and fluorochromes used for flow cytometry analysis.
